# The effect of surgery report cards on improving radical prostatectomy quality: the SuRep study protocol

**DOI:** 10.1186/s12894-018-0403-y

**Published:** 2018-10-19

**Authors:** R. H. Breau, R. M. Kumar, L. T. Lavallee, I. Cagiannos, C. Morash, M. Horrigan, S. Cnossen, R. Mallick, D. Stacey, M. Fung-Kee-Fung, R. Morash, J. Smylie, K. Witiuk, D. A. Fergusson

**Affiliations:** 1 Division of Urology, Department of Surgery, The Ottawa Hospital, University of Ottawa, Ottawa, ON Canada; 2 Ottawa Hospital Research Institute, Ottawa, ON Canada; 3 Faculty of Health Sciences, University of Ottawa, Ottawa, ON Canada; 4 The Ottawa Hospital Cancer Program, Ottawa, Canada

**Keywords:** Prostate cancer, Radical prostatectomy, Surgeon feedback, Surgical report cards, Audit and feedback, Knowledge translation

## Abstract

**Background:**

The goal of radical prostatectomy is to achieve the optimal balance between complete cancer removal and preserving a patient’s urinary and sexual function. Performing a wider excision of peri-prostatic tissue helps achieve negative surgical margins, but can compromise urinary and sexual function. Alternatively, sparing peri-prostatic tissue to maintain functional outcomes may result in an increased risk of cancer recurrence. The objective of this study is to determine the effect of providing surgeons with detailed information about their patient outcomes through a surgical report card.

**Methods:**

We propose a prospective cohort quasi-experimental study. The intervention is the provision of feedback to prostate cancer surgeons via surgical report cards. These report cards will be distributed every 3 months by email and will present surgeons with detailed information, including urinary function, erectile function, and surgical margin outcomes of their patients compared to patients treated by other de-identified surgeons in the study. For the first 12 months of the study, pre-operative, 6-month, and 12-month patient data will be collected but there will be no report cards distributed to surgeons. This will form the pre-feedback cohort. After the pre-feedback cohort has completed accrual, surgeons will receive quarterly report cards. Patients treated after the provision of report cards will comprise the post-feedback cohort. The primary comparison will be post-operative function of the pre-feedback cohort vs. post-feedback cohort. The secondary comparison will be the proportion of patients with positive surgical margins in the two cohorts. Outcomes will be stratified or case-mix adjusted, as appropriate. Assuming a baseline potency of 20% and a baseline continence of 70%, 292 patients will be required for 80% power at an alpha of 5% to detect a 10% improvement in functional outcomes. Assuming 30% of patients may be lost to follow-up, a minimum sample size of 210 patients is required in the pre-feedback cohort and 210 patients in the post-feedback cohort.

**Discussion:**

The findings from this study will have an immediate impact on surgeon self-evaluation and we hypothesize surgical report cards will result in improved overall outcomes of men treated with radical prostatectomy.

**Electronic supplementary material:**

The online version of this article (10.1186/s12894-018-0403-y) contains supplementary material, which is available to authorized users.

## Background

Prostate cancer and the side effects of treatment are major public health issues, since the disease is diagnosed in approximately 18% of men [1]. The vast majority of patients diagnosed currently have localized disease that can be cured surgically with radical prostatectomy [2–4]. Since the prostate is in close proximity to the urethral sphincter and nerves that facilitate erections for sexual function, these structures are often inadvertently injured or purposefully resected during tumour resection. Consequently, approximately 0.3–12.5% of men suffer permanent urinary incontinence after a radical prostatectomy, and up to 30% of men use incontinence pads [5–8]. It is also estimated that 75% of patients will suffer from temporary or permanent erectile dysfunction following a radical prostatectomy [9, 10]. Maintenance of urinary and erectile function is related to surgical technique, and significant differences in function following radical prostatectomy have been observed between surgeons [11–13].

Positive surgical margins occur in approximately 11–38% of patients [14, 15]. A positive surgical margin can be an indication of locally-advanced disease, but is also a reflection of surgical technique. Surgical margin rate has been highlighted as an indicator of surgical quality that should be monitored prospectively [16] Significant variability exists in positive margin rates between surgeons [2, 11, 17, 18]. The surgical margin status of a patient is important because this outcome is highly associated with increased need for secondary salvage treatments and lower overall survival [19–21].

In a 2006 provincial pathology audit, positive surgical margins were found in 33% of patients who had tumours that did not extent outside the prostate (pathologic stage T2) in Ontario, Canada (population 12,160,282). In 2008, a Cancer Care Ontario expert clinician panel set a provincial goal to reduce the incidence of positive surgical margins to less than 25% [22]. Since that time, Cancer Care Ontario has been providing feedback to clinicians on the incidence of positive surgical margins in their patients. As of 2011, the incidence of pT2 positive surgical margins in Ontario had dropped to 21% [23]. This finding supported the hypothesis that prospective reporting and feedback can affect surgeon behaviour, resulting in improvement. The findings in Ontario are consistent with randomized trials that show feedback to clinicians generally result in practice improvement [24].

A potential negative consequence of surgical margin scrutiny by Cancer Care Ontario is that surgeons may be performing a wider excision of peri-prostatic tissue, purposely sacrificing erectile nerve tissue to lower the risk of a positive margin, and as a result compromise urinary and sexual function. Clearly, a balance must be struck between ensuring complete cancer removal and preservation of a patient’s urinary and sexual function. Isolated critical evaluation of surgical margin rates may have had the unintended effect of increasing long term side effects that are important to patients. Ideally, all clinically important outcomes should be assessed when evaluating surgical quality, not only surgical margin status.

The primary purpose of this study is to determine if providing prostate cancer surgeon feedback on their patients’ improves overall surgical quality as measured by surgical margin rates and patient reported functional outcomes.

## Methods

### Study design and setting

This is a prospective cohort quasi-experimental before-and-after study at The Ottawa Hospital. The Ottawa Hospital is an academic hospital affiliated with the University of Ottawa. The Ottawa Hospital serves the Champlain Local Health Integration Network (LHIN), which is a geographic boundary that contains a population of approximately 1.3 million people [25]. Approximately 80% of prostate cancer patients in the Champlain LHIN who receive a radical prostatectomy are treated by one of eight prostate cancer surgeons at the Ottawa Hospital. The intervention in this study is the provision of feedback to prostate cancer surgeons via surgical report cards. These report cards will be distributed every 3 months by email. The report card will present surgeons with outcomes of their patients compared to patients treated by other de-identified surgeons in the study.

### Study participants

All prostate cancer surgeons will participate in this study. While all surgeons will use a common clinical pathway and provide consistent information, the surgical technique is not mandated by the study. All patients undergoing radical prostatectomy by each surgeon will be eligible, regardless of tumour stage, histology and previous pelvic treatments including surgery or radiation. Extent of planned and performed neurovascular bundle preservation will not be mandated, but will be prospectively collected from the operating surgeon. Surgeons will report their nerve spare intent prior to surgery and their perceived nerve spare achieved following surgery. All surgical approaches (open, laparoscopic, and robotic assisted laparoscopic) will be included. Given that patients are contacted for assessment of symptoms, consent will be obtained from each patient. Patients will be excluded if they 1) decline or are incapable of providing consent; 2) are less than 18 years of age; or 3) are being treated outside of The Ottawa Hospital. Patient information and outcomes will be collected prior to surgery and 6- and 12-months following surgery. Approximately 230 radical prostatectomy procedures are performed annually at The Ottawa Hospital.

### Assessment and data collection

#### Pre-surgery

Prior to treatment, patients will complete two functional assessments that were selected based on content validity, common use in clinical practice, and availability in English and French: (1) The Expanded Prostate Cancer Index Composite (EPIC), and (2) the EQ-5D. EPIC is a 50-item robust and comprehensive prostate cancer-specific instrument that measures a broad spectrum of urinary, bowel, sexual, and hormonal symptoms. The EPIC has been widely validated and used in prostate cancer clinical trials and treatment quality initiatives [26] and will serve as the basis for urinary and erectile function assessment. EQ-5D is a simple, easy to use assessment of overall health status and provides a descriptive profile and a single index value for reporting [27]. The EQ-5D is valid and reliable across a broad range of clinical settings and languages.

Other pre-operative baseline characteristics will be collected, including patient age, height, weight, clinical tumour stage, tumour grade, and prostate specific antigen (PSA) concentration. This information is available from the standardized pre-operative pathway documents and will be abstracted by study personnel.

#### Time of surgery

Synoptic reporting will be used by surgeons and pathologists to aid with data collection. Surgeons will document planned and achieved preservation of neurovascular bundles. Pathology reports will be used to determine cancer stage, tumour grade, tumour size, location of extraprostatic tumour extention (if applicable), extent of extraprostatic tumour extension (if applicable), location of a positive surgical margin (if applicable), and extent of a positive surgical margin (if applicable).

#### Post-surgery

At 6 and 12 months post-operative, patients will be mailed the same quality-of-life and functional assessment questionnaires that were completed prior to surgery. Study personnel will contact patients who do not return the questionnaires to facilitate completion or to determine why the patient does not wish to participate. This duration of follow-up was chosen because functional outcomes improve over time and most outcomes have generally stabilized by 12 months post-surgery [28]. Post-operative interventions that could confound results will be determined by asking patients if they currently use erectile function aids, have had urinary continence procedures, received pelvic radiation, or received androgen deprivation. All post-operative PSA values will be documented.

### Surgical report card intervention and control

Descriptive baseline, pathological, and follow-up information will be summarized and tabulated. The primary measures of surgical quality on the surgeon report cards will be: post-operative urinary continence, post-operative erectile function (stratified by baseline function and tumour stage), and rate of positive surgical margins (stratified by tumour stage). An example of a surgical report card is displayed in Additional file 1. Urinary continence will be defined as requiring no continence pads (i.e. score of 0 on question 5 of the EPIC questionnaire), and potency will be defined as a firm enough erection for intercourse (score of 4 on question 18 of the EPIC questionnaire). Changes in patients’ overall health status will also be summarized.

**Fig. 1:**
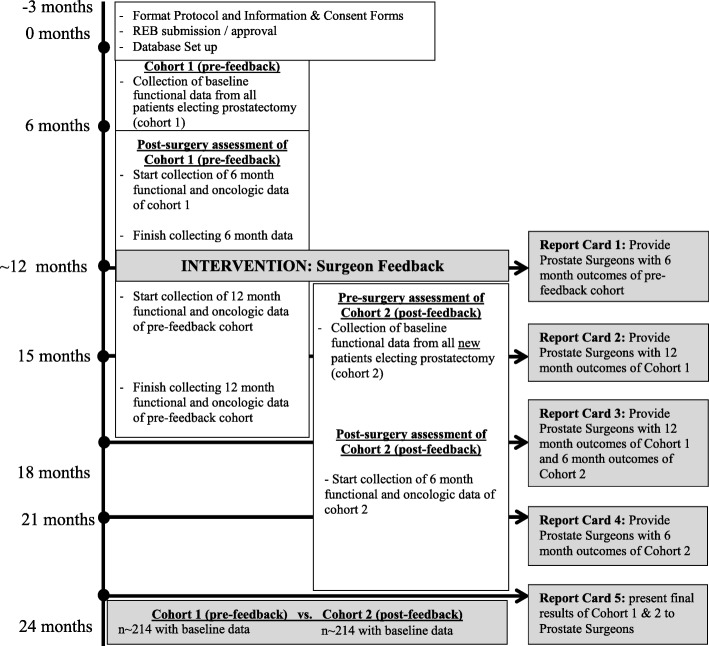
Study timeline

For the pre-feedback cohort, pre-operative, 6-month, and 12-month patient data will be collected but there will be no report cards distributed to surgeons. This will form the pre-feedback cohort. When accrual to the pre-feedback cohort is complete (anticipated to be 12 months from initiation of the study), surgeons will begin receiving report cards. Patients treated after the provision of report cards will form the post-feedback cohort for analyses. A summary of the study timeline is presented in Fig. 1.

### Outcomes

The primary outcomes will be post-operative function of the patient cohort before surgeon feedback (pre-feedback cohort) compared to post-operative function of the patient cohort after surgeon feedback (post-feedback cohort). The secondary comparison will be the proportion of patients with positive surgical margins in the pre-feedback cohort compared to post-feedback cohort. Outcomes will be stratified or adjusted by important co-variates such as patient age, tumour stage, tumour grade, pre-operative PSA, baseline urinary function, baseline sexual function, and nerve preservation status.

### Analysis and sample size

The comparison between pre-and post-feedback functional outcomes will be done by using generalized linear model that treats the intervention as an effect that is either present or absent for a patient (absent for the pre-feedback cohort and present the post-feedback cohort). In the analysis the surgeons will be considered as clusters to account for repeated measurements. In the model other covariates like patient age, tumour stage, tumour grade, pre-operative PSA, baseline urinary function, baseline sexual function, and nerve preservation status will be used. An estimate of the difference between pre-feedback and post-feedback potency or continence will be obtained from the above model.

An overall improvement of 10% in potency or incontinence following the surgeon feedback intervention (post-intervention cohort) will be considered clinically significant. Assuming a baseline potency of 20% and a baseline continence of 70%, 294 patients would be required for 80% power at an alpha of 5%. Assuming as much as a 30% lost to follow up, we will require a minimum of 210 patients in the pre-feedback cohort and 210 patients in the post-feedback cohort.

## Discussion

As a result of improved survival due to early detection of prostate cancer, side effects of radical prostatectomy that affect long term quality-of-life are of paramount importance to patients. However, there are no proven methods to improve surgical quality in these domains. Furthermore, initiatives to lower margin rates may compromise these outcomes if they are not considered. This proposal is innovative and significant because***,*** to the best of our knowledge, it will be the first prospective study that provides feedback to surgeons about oncological and functional outcomes in men treated with radical prostatectomy. Furthermore, it will allow us to assess whether providing feedback to surgeons results in improvement in surgical quality.

The findings from this initiative will have an immediate impact on surgeon self-evaluation, and we hypothesize this will result in improved overall outcomes and satisfaction for men treated with radical prostatectomy at our institution. In addition to the immediate benefits to patient counseling, this study is designed so it can be freely available and transferable for use in any hospital or region that aims to monitor and improve prostate cancer treatment quality.

## Additional file

Additional file 1: Sample of surgical report card. (DOCX 683 kb)
