# Supplementary material for: The effect of surgery report cards on improving radical prostatectomy quality: the SuRep study protocol
Source: BMC Urol. 2018 Oct 19;18:89. doi: 10.1186/s12894-018-0403-y (PMC6194548; doi:10.1186/s12894-018-0403-y)
Supplement: Supplementary file 1 — Sample of surgical report card. (DOCX 683 kb) [file 12894_2018_403_MOESM1_ESM.docx]

**Supplementary Material: Sample of surgical report card**
